# Phylogenetic, Molecular, and Biochemical Characterization of Caffeic Acid *o*-Methyltransferase Gene Family in *Brachypodium distachyon*

**DOI:** 10.1155/2013/423189

**Published:** 2013-01-17

**Authors:** Xianting Wu, Jiajie Wu, Yangfan Luo, Jennifer Bragg, Olin Anderson, John Vogel, Yong Q. Gu

**Affiliations:** ^1^Western Regional Research Center, USDA-ARS, 800 Buchanan Street, Albany, CA 94710, USA; ^2^Department of Plant Sciences, University of California, Davis, CA 95616, USA; ^3^State Key Laboratory of Crop Biology, Shandong Agricultural University, 61 Daizong Avenue, Tai'an, Shandong 271018, China

## Abstract

Caffeic acid *o*-methyltransferase (COMT) is one of the important enzymes controlling lignin monomer production in plant cell wall synthesis. Analysis of the genome sequence of the new grass model *Brachypodium distachyon* identified four COMT gene homologs, designated as *BdCOMT1, BdCOMT2, BdCOMT3,* and *BdCOMT4*. Phylogenetic analysis suggested that they belong to the COMT gene family, whereas syntenic analysis through comparisons with rice and sorghum revealed that *BdCOMT4* on Chromosome 3 is the orthologous copy of the COMT genes well characterized in other grass species. The other three COMT genes are unique to *Brachypodium* since orthologous copies are not found in the collinear regions of rice and sorghum genomes. Expression studies indicated that all four *Brachypodium* COMT genes are transcribed but with distinct patterns of tissue specificity. Full-length cDNAs were cloned in frame into the pQE-T7 expression vector for the purification of recombinant *Brachypodium* COMT proteins. Biochemical characterization of enzyme activity and substrate specificity showed that BdCOMT4 has significant effect on a broad range of substrates with the highest preference for caffeic acid. The other three COMTs had low or no effect on these substrates, suggesting that a diversified evolution occurred on these duplicate genes that not only impacted their pattern of expression, but also altered their biochemical properties.

## 1. Introduction

 Temperate grains like wheat and barley, along with forage grasses, contribute greatly to the human food and animal feed supply. However, the large and complex genomes in these economically important grasses present challenges for genomics studies and map-based cloning of target genes for crop improvement. Similarly, although large perennial grasses like switchgrass and *Miscanthus* are being developed as dedicated herbaceous energy crops, our knowledge about the biological and genetic basis of important bioenergy traits remains limited [1–4]. *Brachypodium distachyon* (hereafter referred as *Brachypodium*) is an attractive experimental system and genomics model for grass research. It has many desirable attributes (small physical stature, short generation time, easy growth requirement, etc.) and numerous freely available genomics resources (high quality genome sequence, EST collection, large-insert BAC libraries, expression/tilling microarray, T-DNA mutant population, etc.) [5]. Thus, *Brachypodium* can serve as a useful model system to address issues unique to grasses ranging from grain improvement to the development of superior bioenergy crops [5–7].

Plant cell walls are a complex composite structure composed of polysaccharides (cellulose and hemicellulose) that are embedded and crosslinked by other polymers and small molecules (lignin, pectin, and ferulic acid). The type of hemicellulose and other components differs significantly between the grasses and dicots [8]. Briefly, primary grass cell walls contain glucuronoarabinoxylans and mixed-linkage glucans as the hemicelluloses, high levels of ferulic acid and *p*-coumaric acids, and the relatively little pectin and protein. Dicot primary cell walls contain xyloglucan, mannans, and glucomannans as the hemicelluloses and high levels of pectin and structural proteins. Secondary cell walls of both grasses and dicots are composed of cellulose, glucuronoarabinoxylans, and lignin. However, the structure of the glucuronoarabinoxylans differs between dicots and grasses. Given the compositional and architectural difference between grass and dicot cell walls, we have turned to *Brachypodium* as our model system to study grass cell walls.

The use of cellulosic biomass crops as a feedstock for the production of transportation fuel offers significant potential environmental and economic advantages. However, due to the difficulty in converting the sugars locked in cellulose and hemicellulose into fuel, our ability to use this renewable feedstock is limited. A deeper understanding of the genes that control cell wall biosynthesis and architecture may allow us to tailor the cell wall to be more amenable to conversion into biofuel [10–14]. Lignin in particular is an impediment to the production of biofuels and forage digestibility [15–20]. Due to crosslinking, it blocks access of cell wall degrading enzymes to cellulose and hemicellulose [21–23]. This makes it necessary to employ harsh pretreatment to disrupt the cell wall structure prior to enzyme hydrolysis. This harsh pretreatment create compounds that are inhibitory to organisms used to convert the free sugars into biofuels like ethanol and butanol. However, due to its phenolic ring structure, lignin is an energy dense compound and thus a high lignin content is desirable if the biomass is used for direct combustion.

Lignins are complex heteropolymers derived from three monolignols, *p*-coumaryl, coniferyl, and sinapyl alcohol. These monolignols produce *p*-hydroxyphenyl (H), guaiacyl (G), and syringyl (S) units, respectively, when incorporated into the lignin polymer. Although composed of only three building blocks, the composition and structure of lignin varies considerably within and among plants because the ratio of monomers varies and there is no repeating linkage structure. Rather, the lignin monomers undergo what appears to be a random cross-linking in the cell wall via several linkages. The S content and the S to G ratio are critical parameters that measure for characterizing lignin composition in the cell wall of angiosperm plants [15, 20]. Enzymes involved in the lignin biosynthesis pathway have been characterized in different plant species [24]. *o*-methylation modulates the chemical and physical properties of the lignin polymer. *o*-methylation mediated by *o*-methyltransferases (OMTs) transfers a methyl group from S-adenosyl-L-methionine (SAM) to the hydroxyl group of a methyl acceptor molecule. Plants contain a large family of OMT enzymes [24]. One of the essential OMTs in lignin biosynthesis is caffeoyl CoA 3-*o*-methyltransferase (CCoAOMT; EC 2.11.104) which is primarily responsible for the initial *o*-methylation of the 3-hydroxyl group and specifically methylates the 3,4-dihydroxy substrate as a CoA-linked thioester. A second important OMT termed caffeic acid *o*-methyltransferase (COMT; EC 2.1.1.68) generally catalyzes the *o*-methylation of the 5-hydroxyl group of 3-methoxy-4,5-dihydroxy precursors. The function of both OMTs has been inferred from the effects on lignin composition through the downregulation of these enzymes in transgenic plants as well as in mutant lines affecting the expression of these genes. For instance, the COMT gene was first cloned in maize by differential screening of a root cDNA library [25]. Characterization of the maize COMT promoter indicated that it directed GUS expression to the xylem and other tissues undergoing lignifications [26]. Transgenic plants with a downregulation of the maize COMT showed a strong decrease of Klason lignin content, a decrease in syringyl units, lower *p*-coumaric acid contents, and the occurrence of an unusual 5-OH guaiacyl unit [27]. These features are similar to those observed in the maize *bm3* (brown-midrib3) mutant lacking the functional COMT gene [27, 28]. Furthermore, it appeared that a decrease in COMT activity led to improved forage digestibility, suggesting that the downregulation of COMT might alters the overall cell wall organization in a way that walls become more accessible to bacterial enzymes [27]. The effects of caffeic acid *o*-methyltransferase (COMT) gene modification on reduction of S monomer level, which is linked directly to the lignin reduction, have been shown in other modified plant species [29–36]. 

 OMT genes have been cloned and characterized in a number of plant species and provide valuable information for the study of the evolution, expression, and function of OMTs in plants [29, 32, 33, 36–40]. However, the characterization of OMT genes in *Brachypodium* has not been reported. In this study, we identified, isolated, and characterized four *Brachypodium* COMT genes. Comparative and phylogenetic analyses revealed that while related grass genomes (rice and sorghum) contain only one copy of COMT, *Brachypodium* possesses four copies located in three different chromosomes. Functional characterization indicated that only the orthologous copy has the biochemical properties similar to other COMTs in grass species although all the *Brachypodium* COMT genes are expressed during plant development. The isolation and characterization of *Brachypodium* COMTs will permit us to develop an efficient system to analyze these enzymes in the lignin biosynthesis pathway through forward and reverse genetics approaches in this tractable model species.

## 2. Results

### 2.1. Genomic Organization of COMT Genes in *Brachypodium*

**Figure 1:**
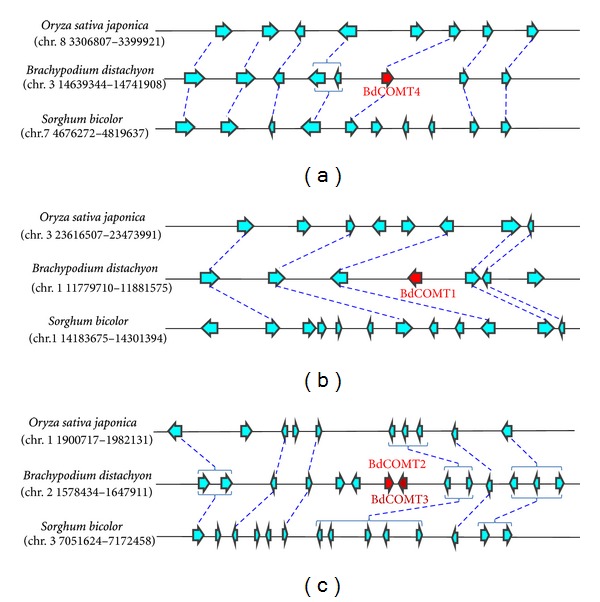
Collinearity analysis of *Brachypodium* COMT gene-containing regions with the orthologous regions of rice and sorghum genomes. Genomic regions harboring *BdCOMT4* (a), *BdCOMT1* (b), *BdCOMT2* (c), and *BdCOMT3* (c) were extracted and the annotated genes in these regions were used to identify orthologous regions from rice and sorghum genomes for collinearity analyses. Annotated genes are represented by arrows with the direction of arrow heads indicating the orientation of transcription. *Brachypodium* COMT genes are labeled in red. Orthologous genes that are shared in two or more genomes are connected with dashed lines. Duplicate genes in a genome are bracketed.

 To identify COMT genes in *Brachypodium*, the rice COMT gene (XP480185) and *Arabidopsis* COMT gene (AAB96879) were used in a BLASTP search against the *Brachypodium* genome database v1.0 annotation (http://www.brachypodium.org/). The top four hits matched annotated *Brachypodium *genes: Bradi1g14870, Bradi2g02380, Bradi2g02390, and Bradi3g16530, with *e*-values lower than 1*e* − 100 (data not shown). These four genes were renamed *BdCOMT1*, *BdCOMT2*, *BdCOMT3*, and *BdCOMT4,* respectively, in this study. From the fifth hit, the *e*-values dropped considerably to be higher than *e* ^−50^ and are, therefore, considered distantly related to COMT. *BdCOMT1* and *BdCOMT4* are located on *Brachypodium* chromosomes 1 and 3, respectively, while both *BdCOMT2* and *BdCOMT3* are located on chromosome 2. Interestingly, the *BdCOMT2* and *BdCOMT3 *genes are next to each other with inverted orientation (Figure 1). When the sequences of the four *BdCOMT* genes were blasted against both the rice and sorghum genome databases, they all retrieved a single strong match to the corresponding COMT gene, suggesting that only *Brachypodium *contains multiple copies of COMT gene. 

 To analyze the genomic organization of these *Brachypodium* genes, genomic regions surrounding the four *BdCOMT* genes were used to search for orthologous regions in the rice and sorghum genomes. The collinearity of these regions was compared to the orthologous regions in the rice and sorghum genomes (Figure 1). The rice and sorghum regions orthologous to the *BdCOMT4* region contained their single COMT genes (Figure 1(a)), indicating that *BdCOMT4* is the orthologous counterpart of the rice and sorghum COMT genes. The rice and sorghum regions orthologous to the other *Brachypodium* COMT gene regions did not contain orthologous COMT genes although the surrounding genes showed general collinearity (Figures 1(b) and 1(c)). This collinearity analysis supports the sequence blast search results indicating that multiple COMT genes exist only in *Brachypodium*.

### 2.2. Phylogenetic Analysis of COMTs in *Brachypodium* and Other Species

**Figure 2:**
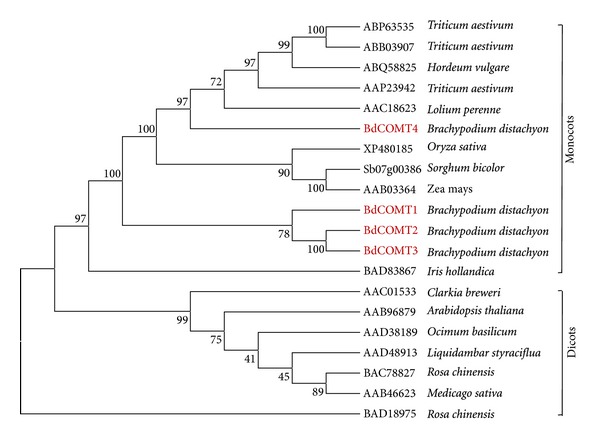
Phylogenetic analysis of COMTs from different plant species. COMT sequences represented by their GenBank or gene IDs from different plant species were extracted from NCBI database and used to construct a phylogenetic tree with phylogenic tree using neighbor-joining method.

 To further confirm the evolutionary relatedness of the *Brachypodium* COMT genes, previously characterized COMTs from both dicot and monocot species were used to perform phylogenic analysis (Figure 2). Dicot COMTs formed one clade and monocot COMTs were grouped into another clade. BdCOMT4 is more closely related to the other grass COMTs than the other three *Brachypodium* COMTs, which formed a distinct clade separated from the orthologous COMT of different grass species. Nevertheless, the observation that BdCOMT1, BdCOMT2, and BdCOMT3 were grouped into monocot COMT clade suggests that duplicated *Brachypodium* COMT genes were generated after the divergence of dicot and monocot.

 We also tested the possibility that BdCOMT1, BdCOMT2, and BdCOMT3 belong to other related *o*-methyltransferases, caffeoyl-CoA *o*-methyltransferase (CCoAOMT) groups, by including a number of characterized CCoAOMTs in our phylogenic analysis. These *Brachypodium* COMTs did not group with CCoAOMTs (see Supplemental Figure  S1 available at http://dx.doi.org/10.1155/2013/423189), providing further supporting evidence that these *Brachypodium * COMT genes are likely to be paralogs of *BdCOMT4* that resulted from gene duplication.

### 2.3. Sequence Analyses of BdCOMTs

 ClustalW analysis was used to align the *Brachypodium* COMT genes at both nucleotide and protein sequence levels. The results showed that they shared over 70% nucleotide identity between any two sequences (data not shown). At the amino acid level, these COMTs shared at least 60% sequence similarity between any two sequences. As expected, BdCOMT2 and BdCOMT3 are the most closely related of the four BdCOMTs. The physical proximity indicated that they are derived from a tandem duplication event (Figure 1(c)). Sequence alignment with COMTs from other species indicated that BdCOMT4 protein shares 58%, 59%, 78%, and 83% sequence similarity with *Arabidopsis thaliana*, *Medicago*, *Zea mays,* and *Oryza sativa* COMT proteins, respectively.

**Figure 3:**
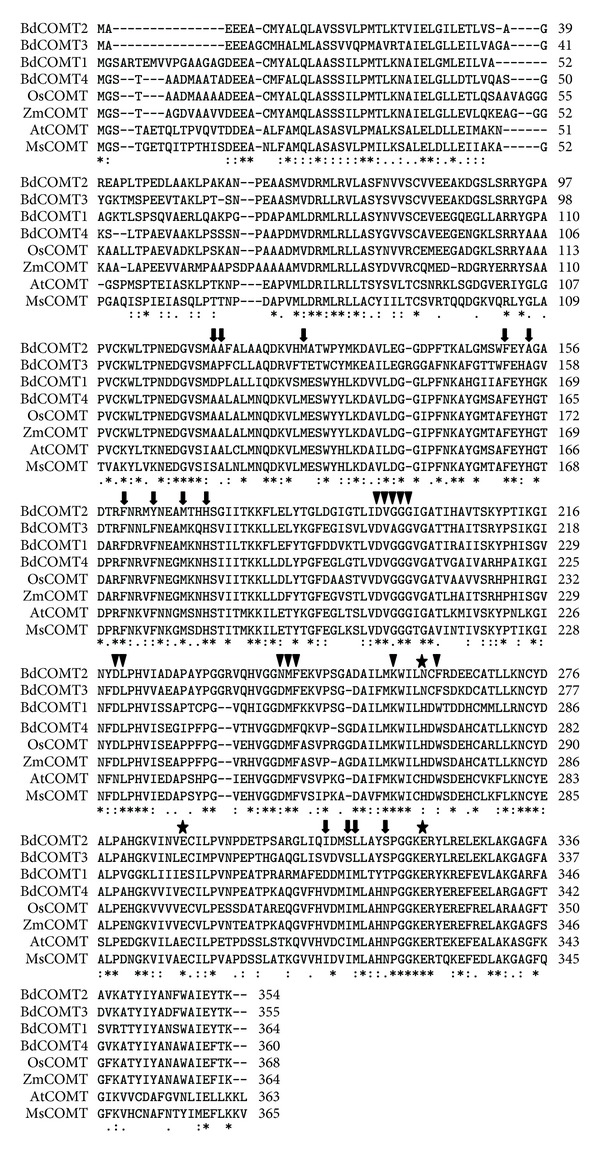
Comparison of amino acid sequences of four BdCOMTs (BdCOMT1, BdCOMT2, BdCOMT3, and BdCOMT4) with COMTs from *Oryza sativa* (OsCOMT)*, Zea mays* (ZmCOMT)*, Arabidopsis thaliana* (AtCOMT), and* Medicago sativa* (MsCOMT). Inferred from MsCOMT crystallographic structure, substrate binding residues are indicated by arrows, methyl donor binding sites by triangles, and catalytic residues by stars. An asterisk “∗” indicates identical residues in all sequences. A semicolon “:” indicates strongly conserved residues (score >0.5), and a dot “.” indicates weaker conserved residues (score <0.5) [9].

From examination of crystal structure of the alfalfa COMT protein, amino acids responsible for catalytic sites and substrate binding sites have been inferred [41]. There are three catalytic sites predicted to be conserved within COMT genes. Two catalytic sites, E297 and E329, in MsCOMT are conserved among all four BdCOMT proteins (Figure 3). The other catalytic site H269 in MsCOMT is conserved in BdCOMT4 (H266) and BdCOMT1 (H270). However, in BdCOMT2 and BdCOMT3, it was replaced with N at this site. Thirteen substrate binding sites have been previously predicted based on MsCOMT crystal structure; twelve of these sites are conserved in BdCOMT4. The only difference in sites is a change at I316 in MsCOMT to V313 in BdCOMT4. It seems this mutation occurred in MsCOMT since OsCOMT, ZmCOMT, and AtCOMT all encode V at this position. However, this site was changed to D in BdCOMT1, and it was mutated to encode I in BdCOMT2. In comparison with BdCOMT4, more changes in these substrate binding sites were observed in BdCOMT2 (8 sites), BdCOMT3 (8 sites), and BdCOMT1 (7 sites). Thus, although BdCOMTs have an overall higher amino acid similarity among themselves when each is compared to MsCOMT, BdCOMT4 and MsCOMT showed higher conservation in the substrate binding sites. The methyl donor (SAM) binding sites were also analyzed. Among all five donor binding regions, there is only one site (W271 in MsCOMT) where BdCOMT2 and BdCOMT3 are similar to each other but different from other COMTs. Otherwise, all sites are generally conserved among BdCOMTs and COMT genes from other species (Figure 3). 

### 2.4. Tissue-Specific Expression of BdCOMT Genes

**Figure 4:**
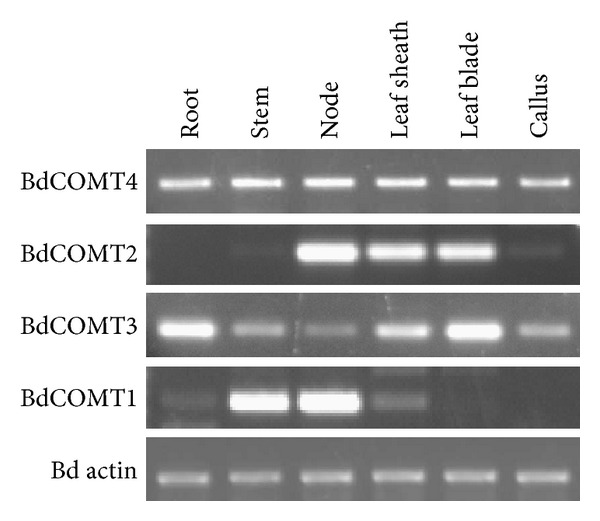
Tissue-specific expression of BdCOMT genes. Total RNAs were extracted from different *Brachypodium* tissues as indicated and used for synthesis of cDNA for RT-PCR. Gene-specific primers for each *Brachypodium* COMT genes were designed and used to amply corresponding transcripts. Primers specific for actin expression are used as the internal control to indicate that equal amount of cDNAs was used in each RT-PCR assay.

 PCR primers specific for each *Brachypodium* COMT gene were designed to characterize the expression of these genes in various tissues including root, stem node, stem internode, leaf sheath, leaf blade and callus (Figure 4). In this semiquantitative RT-PCR analysis experiment, *BdCOMT4* was found to be expressed uniformly and constitutively in all the tissues examined as compared to the ubiquitous *Brachypodium* actin gene expression. In contrast, the other three COMT genes showed very different expression patterns. For example, *BdCOMT2* was only expressed in the internode, leaf sheath and leaf blade, but absent in the other tissues, whereas *BdCOMT3* appeared to be expressed in all the tissues with the highest expression level detected in root and leaf blade, suggesting that these tandemly duplicated genes are now regulated differently at the transcriptional level. *BdCOMT1* gene was strongly expressed in the stem node and internode but barely detected in the other tissues (Figure 4). 

### 2.5. Expression and Biochemical Characterization of Recombinant BdCOMT Proteins

**Figure 5:**
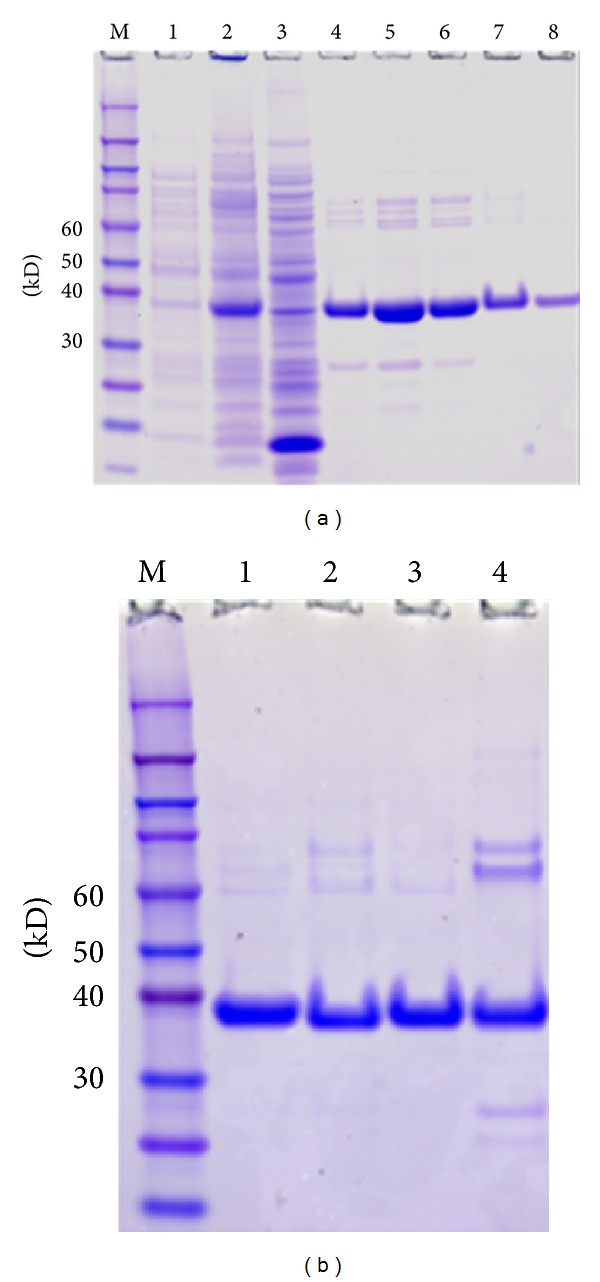
SDS-PAGE gel analysis of purification of recombinant BdCOMTs in *E. coli*. (a) Expression and purification of BdCOMT4 in *E. coli*. Lane M, protein size standards. Lane 1, total lysate of BL21(DE2) pLysS *E.coli.* containing pQE-BdCOMT1 before IPTG induction. Lane 2, total lysate of BL21(DE3)pLysS *E. coli.* containing pQE-BdCOMT1 with 1 mM IPTG induction for 4 hrs. Lane 3, flow-through lysate. Lanes 4–8, BdCOMT1 protein samples collected in different 100 *μ*L fractions after adding the elution buffer. (b) SDS-PAGE gel analysis of purified BdCOMT proteins. Lane M, Protein size standards. Lane 1, 3 *μ*g BdCOMT4; Lane 2, 3 *μ*g BdCOMT2; Lane 3, 3 *μ*g BdCOMT3; Lane 4, 3 *μ*g BdCOMT1.

The gene expression analysis indicated that all the *Brachypodium* COMT genes are active genes. To biochemically characterize these *Brachypodium* COMTs, full-length cDNA for each COMT gene was isolated and cloned in frame into His-tagged pQE-T7 expression vector. The recombinant proteins were expressed in *E. coli* and purified with nickel-conjugated agarose beads (Figure 5(a)). Minor nonspecific protein contamination was detected in purified recombinant proteins despite imidazole concentrations in the wash series up to 50 mM (Figure 5(b)). Nevertheless, we estimated that the purified proteins reached at least 85% purity. The purified recombinant proteins were used for enzymatic activity assays with S-[methyl- ^14^C]-adenosyl-L-methionine (SAM) as the methyl donor and six substrates including caffeic acid, caffeoyl aldehyde, and four flavonoid compounds (Table 1). Among these substrates, BdCOMT4 showed the highest activity on caffeic acid, and this activity was set as 100% for comparison with other substrates. BdCOMT4 activity on caffeoyl aldehyde was less than half of that on caffeic acid (41.1%). BdCOMT4 also showed high catalytic activity on luteolin (85.0%). This flavonoid compound was a preferred substrate for rice COMT [38]. In addition, BdCOMT4 displayed activity on three other flavonoid substrates at different levels, ranging from 17.0 to 31.1% (Table 1). These results suggest that BdCOMT4 prefers the most likely *in vivo* substrate, caffeic acid, but is also active on a range of substrates. Kinetic parameters of purified recombinant BdCOMT4 were determined with caffeic acid as the substrate. The apparent *K*_*m*_ and *V*_max⁡_ values calculated based on the nonlinear Michaelis-Menten plot were 187 *μ*M and 44 nkat/mg protein, respectively. Compared to the kinetic parameter of rice COMT on caffeic acid [38], BdCOMT4 has a higher *K*_*m*_ (rice COMT; *K*_*m*_ 69 *μ*M) and a greater *V*_max⁡_ (rice COMT; *V*_max⁡_ 5.5 nkat/mg protein). In contrast to BdCOMT4, the three other purified *Brachypodium* COMTs failed to show significant enzymatic activity on the substrates examined (Table 1). Further studies will be required to determine if these proteins possess enzymatic activity with different substrates. However, based on sequence similarity and phylogenetic analysis result, currently, we still classified them as COMT genes in this study. 

## 3. Discussion

To characterize COMT in the model grass *Brachypodium*, four COMT genes were identified at different locations in three chromosomes. Phylogenetic analysis suggests that they can be grouped into the COMT family. Comparative analysis indicated that BdCOMT4 is the orthologous copy of COMT genes in the rice and sorghum, while the other three *Brachypodium* COMT genes do not have orthologous copies in rice and sorghum genomes, suggesting that they are unique to *Brachypodium *(Figure 1). Phylogenetic analysis also indicated that they do not belong to CCoAOMTs, a different type of OMT closely related to COMT [24]. Therefore, BdCOMT4 is the ancestral COMT gene since its counterpart is present in rice and sorghum. The other *Brachypodium* COMT genes are likely to be paralogs of BdCOMT4, resulting from gene duplications and translocations into different locations. It has been shown that gene duplication followed by translocation often results in the presence of noncollinear genes in syntenic regions of two closely related genomes [42]. The fact that both rice and sorghum do not have these paralogs suggests that it is most likely that the gene duplications occurred after *Brachypodium* lineage diverged from the rice and sorghum lineages. Evolutionarily, *Brachypodium* is more closely related to wheat and barley since they both belong to the subfamily Pooideae. Rice belongs to the subfamily Ehrhartoideae and sorghum to the subfamily Panicoideae [5]. Collinearity analyses of *Brachypodium* with wheat and barley are very limited because their genome sequences are not available yet. Since wheat is polyploid species, we searched diploid barley EST collection for expressed COMT transcripts. Phylogenetic tree analysis indicated the coding regions of four barley EST contigs aligned with four *Brachypodium* COMT genes (Supplemental Figure S2), suggesting both *Brachypodium* and Triticeae developed multiple COMT genes. However, a direct orthologous relationship among the four BdCOMTs and the four barley genes cannot be not determined here.

On the other hand, we also cannot exclude the possibility that COMT gene duplications occurred prior to the divergence of the *Brachypodium*, rice, and sorghum genomes, and duplicate paralogs were then deleted in the rice and sorghum lineages, resulting in their different evolutionary fates. The different fates of duplicated genes in different species can be explained by the birth-death evolution model in which new genes are created by gene duplication and some of these duplicate genes stay in the genome over time, whereas others are inactivated or deleted from the genome [43]. 

 The birth-death model provides a plausible explanation for the evolution of the OMT gene family. Plant OMTs constitute a large family of enzymes that methylate the oxygen atom of a variety of secondary metabolites including phenylpropanoids, flavonoids, and alkaloids [24]. These enzymes play a key role in lignin biosynthesis, stress tolerance, and disease resistance in plants. Duplication of OMT genes provides raw materials for diversified evolution by mutations to create new OMT genes with different structures and functions. The four *BdCOMT* genes and those barley COMTs could represent a process of diversified evolution of duplicate genes in *Brachypodium*. These duplicate *Brachypodium* genes now display different expression patterns and tissue specificities in development compared to the ancestral *BdCOMT4* (Figure 4). In addition, sequence analysis indicated that the amino acids responsible for enzyme catalytic activity and donor binding are highly conserved in these proteins. Less conservation was observed in substrate binding sites (Figure 3), suggesting that they have evolved or are still evolving to have altered substrate binding properties. This notion is in agreement with the low or lack of activity of these proteins on the common COMT substrates (Table 1). Genes with high sequence similarity, but distinct substrate specificity, have been observed in the OMT gene family [33, 44]. Due to the changes in substrate specificity, they are often reclassified into different OMT classes [33]. Although the preferred substrates for these three *Brachypodium* COMTs were not identified in this study, the expression and conservation of amino acids responsible for enzyme activity suggest that they are likely to be active genes. The differential expression pattern in different tissues might suggest that these genes might have different roles in plant development. Further characterization of BdCOMT1, BdCOMT2, and BdCOMT3 by examining more substrates or gene silencing through transgenic approaches is needed to understand their functions in *Brachypodium*. It is also interesting to note that *Brachypodium* has the most compact genome of all the species examined and it tends to have fewer members within gene family [6]. These facts suggest that the BdCOMT1-3 have been retained because they might have adopted some other functions. 

Enzymatic assays of the *Brachypodium* COMTs revealed that BdCOMT4 had strong COMT activity on caffeic acid and caffeoyl aldehyde substrates with > twofold preference for caffeic acid over caffeoyl aldehyde (Table 1). In addition, BdCOMT4 showed high activities on several flavonoid compounds. These results indicate that BdCOMT1's substrate specificity is quite similar to that of rice COMT [38], but differs from the specificity of the COMT (MsCOMT) from alfalfa, a dicot species. MsCOMT exhibited the highest activity on 5-hydroxyconiferaldehyde and caffeoyl aldehyde and the lowest activity on caffeic acid [45]. Low activity on caffeic acid substrates was also observed for COMTs from other dicots such as sweetgum and *Vanilla planifolia* [45–48]. In *Vanilla planifolia*, Van COMT showed higher preference for caffeoyl aldehyde to caffeic acid as a substrate. In addition, Van COMT has low activity on 1,2,3-trihydroxybenzene derivatives such as propyl gallate and methyl gallate, but these two substrates were preferred by Van OMT-2 and Van OMT-3, which are closely related to Van COMT at the sequence level [33]. Interestingly, BdCOMT4 also showed considerable activity on flavonoid substrates such as propyl gallate and methyl gallate substrates, although the most preferred substrate is caffeic acid (Table 1). A similar preference for these different types of substrates was also observed in rice COMT [38]. Whether rice COMT and BdCOMT4 have roles in pathways involved in flavonoid compound biosynthesis is not known. It appears that the substrate preference for COMTs might even not be shared by the monocot species. It has been shown that several monocot COMTs have substrate preference for aldehydes over alcohols and acids such as ryegrass and sorghum [49, 50]. It is also interesting to note that the expression pattern of COMT is different among monocot species. For example, while BdCOMT4 and wheat COMT have a similar expression level in different tissues [51], COMT from maize is highly expressed only in roots [25]. COMT from ryegrass is abundantly expressed in stems [52]. Whether the differential expression of COMTs in different species suggests that they may serve additional function in some species in comparison to others is also not clear. 

 The difference in substrate specificity among COMTs from different plant species is intriguing. Several reports have demonstrated that a few or single amino acid changes can drastically alter substrate specificity among plant OMTs [35, 44]. Structure analysis of alfalfa COMT by crystallography suggests motifs or amino acids critical for enzyme activity on substrates [41]. Alignment of BdCOMT4 with COMTs from other species including alfalfa revealed high conservation on these amino acid sites; the change from I316 in MsCOMT to V313 in BdCOMT4 marks the only difference among the 13 active substrate binding sites (Figure 3). Interestingly, *Arabidopsis* COMT has valine at the corresponding position (Table 1) and its activity on caffeic acid is higher than that on caffeoyl aldehyde substrate [53]. In addition, this isoleucine in MsCOMT is changed to leucine with a similar amino acid structure in Van COMT in *Vanilla planifolia*. The substrate preference of Van COMT is also similar to MsCOMT with caffeoyl aldehyde as a more highly preferred substrate than caffeic acid [33]. The question of whether a single amino acid change at this site caused the difference in the substrate specificity toward caffeic acid and caffeoyl aldehyde among these COMTs merits further investigation. As we understand more of the effects of specific amino acids on enzyme activity, it may someday become possible to engineer COMT that can act on a particular substrate of interest. 

Despite the importance of COMT in lignin biosynthesis, extensive characterization of COMTs has mainly been reported on dicot species such as *Arabidopsis* and alfalfa. Recent studies on the characterization of monocot COMTs using both biochemical and gene silencing approaches have greatly facilitated a better understanding of their functions in lignin biosynthesis in grass species [27, 38, 39, 49, 54]. Lignin content and composition are known to differ among plant species, contributing to the architecture difference of cell walls between dicots and monocots [8]. Several monocot species such as switchgrass and *Miscanthus* are targeted for development into an herbaceous biomass fuel crops. However, those dedicated biofuel crops are often not ideal for basic research due to their large physical stature and complex genome structure. The demonstration of many favorable characteristics of *Brachypodium* as a desirable experimental system promised to provide unique knowledge on grass biology including the lignin biosynthesis pathway. The notion that *Brachypodium* and *Miscanthus* have similar cell wall compositions further supports the use of *Brachypodium* as a model for investigating grass cell walls [55]. In this study, we showed that, by evolution, BdCOMT4 is an ortholog of COMTs in grasses including rice-and-sorghum-based gene collinearity analysis. It may also share similar biochemical and functional properties with COMTs in other grass species. Thus, knowledge gleaned from cell wall biology in *Brachypodium *could greatly benefit grass bioenergy crop research for sustainable fuel production. 

## 4. Materials and Methods

### 4.1. Plant Materials

*Brachypodium* seeds (accession Bd21-3) were sown in a soilless mix (Supersoil, Rod McLellan Co., Marysville, OH, USA), placed in 4°C cold room in dark for a two-week vernalization period, and then transferred to a greenhouse with temperature range of 24°C in the day and 18°C at night with supplemental lighting to extend day length to 16 hours. Plant tissues (leaf blade, leaf sheath, stem node, stem internode, and root) were collected 3-4 weeks after growth in the greenhouse and frozen in liquid nitrogen for mRNA extraction. 

### 4.2. Identification of *Brachypodium* COMT Genes and Collinearity Analysis

The rice and *Arabidopsis* COMT gene sequence were used in a BLASTN search against the *Brachypodium* genome database. Four hits matched annotated *Brachypodium* genes Bradi3g16530, Bradi2g02380, Bradi2g02390, and Bradi1g14870. These genes were designated *BdCOMT4*, *BdCOMT2*, *BdCOMT3*, and *BdCOMT1*, respectively. Collinearity analysis was performed using CoGe's GEvo tool (http://genomevolution.org/CoGe/index.pl/). Genomic regions (~100 kb in size) surrounding *Brachypodium* COMT genes were analyzed by comparison with orthologous regions in rice and sorghum. Collinear genes present in more than two genomes were identified based on BLASTN algorithm with an *e*-value of 1*e* − 30. Noncollinear gene sequences that are present in one genome, but absent in the other genomes, were also marked.

### 4.3. Protein Sequence Alignment

 COMT protein sequences of alfalfa, *Arabidopsis*, maize, and rice were collected from the NCBI database. The four *Brachypodium* COMT protein sequences annotated in the genome database were confirmed with the full-length cDNA sequences amplified from *Brachypodium* total mRNA (see below). A total of eight COMT protein sequences were aligned with ClustalW2 (multiple sequence alignment tool) (http://www.ebi.ac.uk/Tools/msa/clustalw2/). Predicted substrate binding sites, methyl donor binding sites, and catalytic sites were inferred according to alfalfa COMT crystal structure analysis [41].

### 4.4. Phylogeny Analysis

 The coding sequences of the four *Brachypodium* COMT genes were used to construct a phylogeny tree with available COMT sequences from different plant species by MEGA5 program with the neighbor-joining method. The evolutionary history was inferred using the neighbor-joining method [56]. The bootstrap consensus tree inferred from 1000 replicates was taken to represent the evolutionary history of the taxa analyzed [57]. Branches corresponding to partitions reproduced in less than 50% bootstrap replicates were collapsed. The percentage of replicate trees in which the associated taxa clustered together in the bootstrap test (1000 replicates) were shown next to the branches [57]. All positions containing gaps and missing data were eliminated from the dataset for analysis. 

### 4.5. RT-PCR

Plant tissues were ground in liquid nitrogen and extracted with TRIzol Reagent (Invitrogen, Inc.). Total mRNA was quantified by ND-1000 Spectrophotometer (NanoDrop Technology, Inc.). cDNA was synthesized by the SuperScript III kit (Invitrogen, Inc.). PCR primers specific for each *Brachypodium* COMT gene were designed as below: BdCOMT1: forward, 5′-GAGGTCGAGGAGGGGCAGGAG-3′, reverse, 5′-CGCCAACATCCACGAGGGTCTT-3′. BdCOMT2: forward, 5′-TGAGCGCCGGCAGGGAAGC-3′, reverse, 5′-T GGCCTCGTTGTACATGCGGTTGA-3′; BdCOMT3: forward, 5′-CGTCTCCATGGCCC CCTTCTG-3′, reverse, 5′-CCGGCGACATCCACGAGGAC-3′; BdCOMT4: forward, 5′-CCGCCCTCGCGCTCATGA A-3′ reverse, 5′-ACGTGGGGGAGGTCG AAGTTGAT-3′; The *Brachypodium* actin gene served as an expression control for RT-PCR. The primer set for amplifying its transcript is as follow: forward: 5′-AAGTACCCTATTGAGCATGG-3′; reverse: 5′-CGTAGTCAAGAGCCACATATG-3′.

### 4.6. cDNA Isolation and Expression Vector Cloning

 Full-length cDNA of each BdCOMT gene was amplified by gene-specific primers. To facilitate cloning, NdeI and XhoI restriction sites were added at the beginning of 5′ primers and in the end of 3′ primers, respectively. Amplified fragments were cloned in frame at the *NdeI* and *XhoI* sites into pQE-T7-1 vector (Qiagen, Inc.). Cloned plasmids were named as pQE-BdCOMT1, pQE-BdCOMT2, pQE-BdCOMT3, and pQE-BdCOMT4. Each plasmid was sequenced to confirm the sequence accuracy and then retransformed into *E. coli* Bd21 (DES) strains (Invitrogen, Inc.) for protein expression.

The cloning primers for each COMT gene are listed below: *BdCOMT4*: forward, 5′-GGAATTCCA TATGAAACAGATGGGTTCCACGGCGGCGGA-3′; reverse, 5′-CCGCTCGAGTCTTACTACTACTTG GTGAACTCGATGG-3′; *BdCOMT2*: forward, 5′-GGAATTCCATATGAAACAGATGGCCGAGGA GGAGGCGTG-3′; reverse, 5′-CCGCTCGAGTCTTACTACTACTTGGTGTACTCGATGGCCCAGA A-3′; *BdCOMT3*: forward, 5′-GGAATTCCATATGAAACAGATGGCGGAAGAGGAAGCCGGGTG-3′; reverse, 5′-CCGCTCGAGTCTTACTACTACTTGGTGTACTCGATGGCCCAGAA-3′; *BdCOMT1*: forward, 5′-GGAATTCCATATGAAACAGATGGGCTCTGCCCGCACCG-3′; reverse, 5′-CCGCTC GAGTCTTACTACTATTTGGTGTACTCAATGGCCCAGGAG-3′.

### 4.7. Protein Expression, Purification, and Quantification

A single *E. coli* clone carrying the expression construct was inoculated into 6 mL Luria broth (LB) medium with 50 *μ*g/mL kanamycin and incubated in 37°C shaker overnight. The overnight culture (1 mL) was added to 500 mL (1 : 500 ratio) LB medium with 50 *μ*g/mL kanamycin. When OD 600 of the culture reached 0.7, IPTG was added to the final concentration of 0.2 mM to induce protein expression and incubated at room temperature for 16 hours with shaking.

 Cells were harvested and suspended in 30 mL lysis buffer (50 mM NaH_2_PO_4_, 300 mM NaCl, and 10 mM imidazole). A lysozyme was added to the final concentration of 1 mg/mL and the mixture was placed on ice for 30 min before sonication. *E. coli* soluble fraction was collected after centrifugation and incubated with 1 mL Ni-NTA agarose beads (Qiagen) by shaking at room temperature for 1 hour. Beads were washed three times in a column each with 50 mL lysis buffer containing imidazole with step increase of concentration at 20 mM, 30 mM, and 50 mM. Proteins were eluted by elution buffer (50 mM NaH_2_PO_4_, 300 mM NaCl, 200 mM imidazole, and 0.05% Tween 20) and stored at −20°C following addition of glycerol to a final concentration of 30%. Proteins were quantified by the BCA assay kit (Thermal Scientific, Inc.). 

### 4.8. Enzyme Activity Assay

Assay reaction mixture contained 5 mM substrate, 3 *μ*g COMT protein, 0.5 mM S-[methyl-^14^C]-adenosyl-L-methionine (SAM) (47 mCi mmol^−1^), 0.1 M Tris-HCl (pH = 7.5) in a final volume of 50 *μ*L reaction system. After 20-minute incubation at 30°C, the reaction was stopped by adding 50 *μ*L 0.2 M HCl and 200 *μ*L hexane : ethyl acetate (1 : 1 ratio) mixture to extract methylated substrate. 20 *μ*L of the supernatant layer containing ^35^S-labeled methylated products from the reaction mixture was moved into 2 mL cocktail solution for liquid scintillation counting by LS6500 Scintillation System (Beckman Coulter, Inc.). Each assay was performed with three replicates and repeated three times. Control reaction was performed in the same manner without addition of COMT protein.

### 4.9. Determination of *K*_*m*_ and *V*_max⁡_ Value of BdCOMT1 Protein

 Steady kinetic curves were determined using different caffeic acid substrate concentrations under the same reaction conditions as described in the enzyme activity assay except 3 *μ*L [^14^C]SAM and 10 *μ*L of unlabeled S-adenosyl-L-methionine (SAM) (Sigma, Inc.) were used. The substrate concentrations in this array were 0.05, 0.1, 0.25, 0.5, 1.0, and 2.0 mM. The *K*_*m*_ and *V*_max⁡_ were calculated from the nonlinear Michaelis-Menten plot enzyme activity assay by Prism 5 for Windows (GraphPad Software, Inc.). The counts per second were calculated to nanomoles of product produced per second (nkat), based on the specific activity (SA) of the ^14^C-labeled substrate and the disintegrations per Minute (DPMs) read by the scintillation counter.

## Supplementary Material

Supplemental Figure 1: Phylogenetic analysis of COMT and CCoAOMT genes among species
Supplemental Figure 2: Phylogenetic analysis of BdCOMT and barley COMT ESTs

## Figures and Tables

**Table 1:** Relative activity (%) of BdCOMT1 recombinant protein purified with six substrates. (^a^34 nkat mg^−1^).

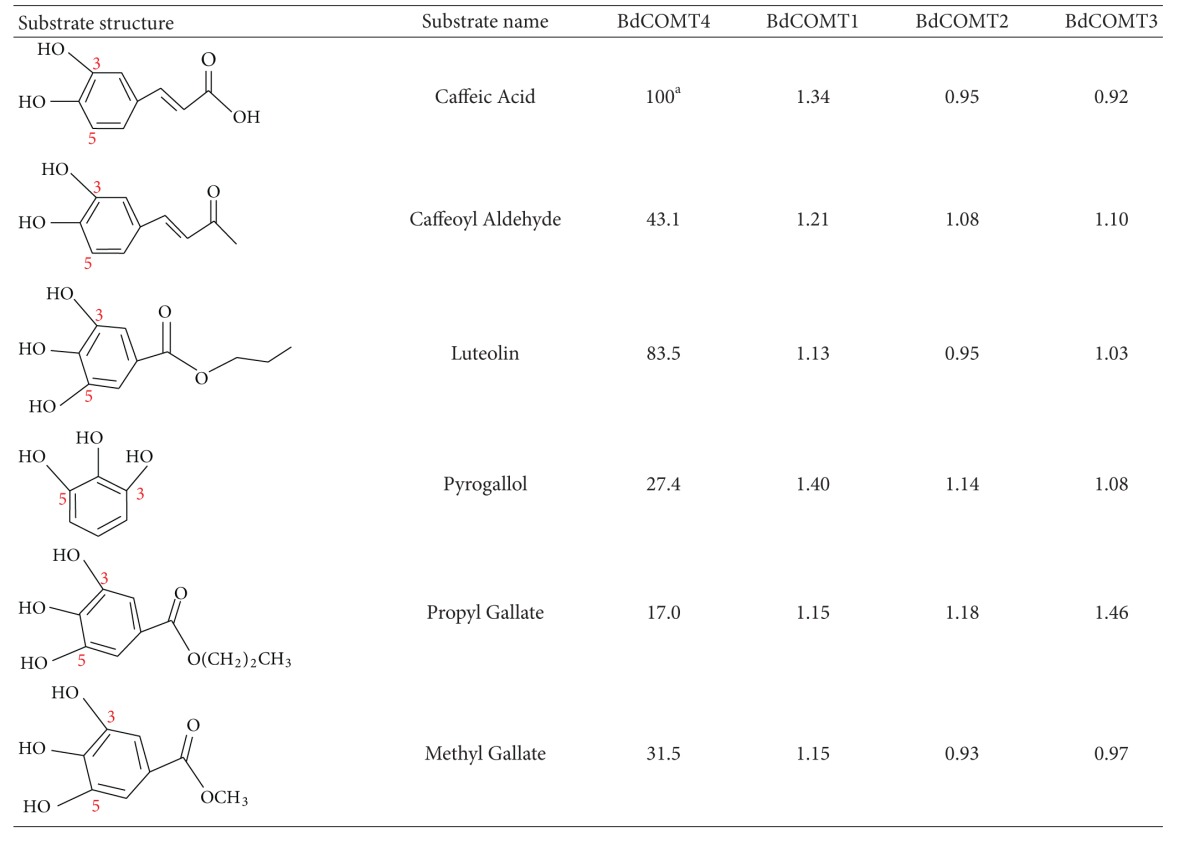

## Abbreviations

CCoAOMT: Caffeoyl CoA 3-*o*-methyltransferase
COMT: Caffeic acid *o*-methyltransferase
PCR: Polymerase chain reaction.
