# Supplementary material for: Phylogenetic, Molecular, and Biochemical Characterization of Caffeic Acid o-Methyltransferase Gene Family in Brachypodium distachyon
Source: Int J Plant Genomics. 2013 Jan 17;2013:423189. doi: 10.1155/2013/423189 (PMC3562662; doi:10.1155/2013/423189)
Supplement: Supplementary file 1 — Supplemental Figure 1: Phylogenetic analysis of COMT and CCoAOMT genes among species Supplemental Figure 2: Phylogenetic analysis of BdCOMT and barley COMT ESTs [file 423189.f1.docx]

**Supplemental Figure 1: Phylogenetic analysis of COMT and CCoAOMT genes among species**

The Neighbor-Joining tree shows that BdCOMT1 is grouped into the major clades of COMTs with other grass species. The other 3 BdCOMT genes are grouped into individual clades separately from BdCOMT1 but still remained in the major COMT cluster but not CCoAOMT clusters. Group A1,A2, B1,B2 and B3 classification is the phylogenetic lineages identified within the O-methyltransferase (OMT) family (Lam et al., 2007).

**Supplemental Figure 2: Phylogenetic analysis of BdCOMT and barley COMT ESTs**

Barley EST collection was blasted using BdCOMTs and significantly matched ESTs were extracted and assembled into contigs. The EST contigs are named by the final contig after trimming of chimeric EST, removing low quality ends from poorly trimmed sequences, and manually editing the assembly. Individual EST ( e.g. AK359402) that is not assembled into any contig due to single nucleotide polymorphism was also included in the analysis. The number of ESTs forming that contig is indicated. For example, contig30 23r indicates that there are 23 ESTs in contig30. Both coding (cod) and protein sequences were used in phylogenetic tree analyses. BRADI3G16530, BRADI2G02380, BRADI2G02390, and BRADI1G14870 represent BdCOMT4, BdCOMT2, BdCOMT3, and BdCOMT1, respectively.
